# Supplementary material for: Persistent accelerations disentangle Lagrangian turbulence
Source: Nat Commun. 2019 Aug 7;10:3550. doi: 10.1038/s41467-019-11060-9 (PMC6685982; doi:10.1038/s41467-019-11060-9)
Supplement: Supplementary file 1 — Supplementary Information [file 41467_2019_11060_MOESM1_ESM.pdf]

Supplementary Information for
"Persistent accelerations disentangle Lagrangian turbulence"

Bentkamp et al.

(Dated: July 3, 2019)

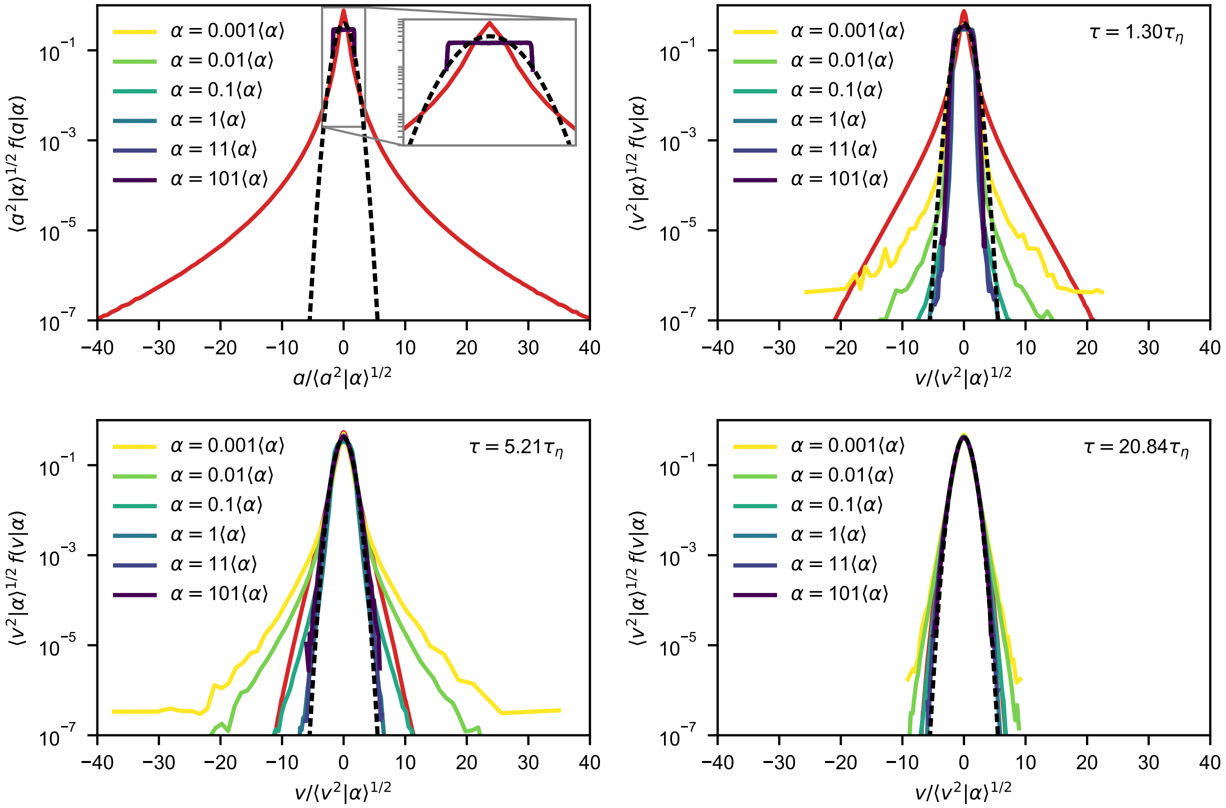

Supplementary Figure 1. **Conditional acceleration and velocity increment PDFs** for $\Theta = 0\tau_\eta$ at $R_\lambda \approx 350$. Conditional PDFs are shown as colored lines and their unconditional counterpart as a red line. A Gaussian PDF is plotted for comparison (black, dashed line). Without the coarse-graining, conditional acceleration PDFs (upper left panel) reduce to uniform distributions. Conditional increment PDFs for small time lags still resemble this distribution but are smoothed out (upper right panel). The conditional increment PDFs for small α display heavy tails, in particular for intermediate time lags (lower left panel). Compared to the main text, we choose more extreme values of α because the distribution of α is wider for $\Theta = 0\tau_\eta$.

SUPPLEMENTARY NOTE 1. CHOICE OF THE COARSE-GRAINING TIME SCALE

Our framework requires a reasonable choice of the coarse-graining time scale Θ, which is the only free parameter in the theory. Intuitively, the coarse-grained squared acceleration is intended to capture the intensity of small-scale turbulence a given particle is subjected to. Hence, we need to choose Θ large enough to even out fluctuations within the same small-scale structure, but also small enough to avoid mixing different regions of the flow. This is why we look for values on the order of the Kolmogorov time scale τ_η, the characteristic time of the small scales in turbulence. More concretely, we look for a choice of Θ that renders the conditional PDFs Gaussian on all scales. Here we elaborate our choice of Θ and show how the results depend on it.

First of all, we consider the case without coarse-graining, i.e. conditioning on the instantaneous squared acceleration $\mathbf{a}^2(t)$, which is equivalent to letting $\Theta \to 0$. The resulting conditional increment and acceleration PDFs are shown in Supplementary Fig. 1. They deviate significantly from Gaussian PDFs, in particular on small scales. The conditional acceleration PDF even reduces to a uniform distribution, a result that can also be shown analytically. This shows that the naive approach of conditioning directly on $\mathbf{a}^2(t)$ does not yield the desired decomposition of the flow. A coarse-graining is needed to decouple the decomposition procedure from instantaneous fluctuations of the acceleration. For comparison, we also show the statistics predicted by the framework at $\Theta = 0$ in Supplementary Fig. 2. The acceleration and increment PDFs are indeed too heavy-tailed compared to the DNS data. However, the fact that the deviations are not very strong shows that the framework is generally robust with respect to the choice of the coarse-graining time scale.

Next, we evaluate results for varying Θ. In order to reduce the number of observables that need to be considered, we focus on acceleration statistics. The reason is that increment statistics are ultimately simply the transition between the small-scale limit, i.e. the acceleration PDF (up to rescaling), and the large scale-limit, i.e. the velocity PDF (up to

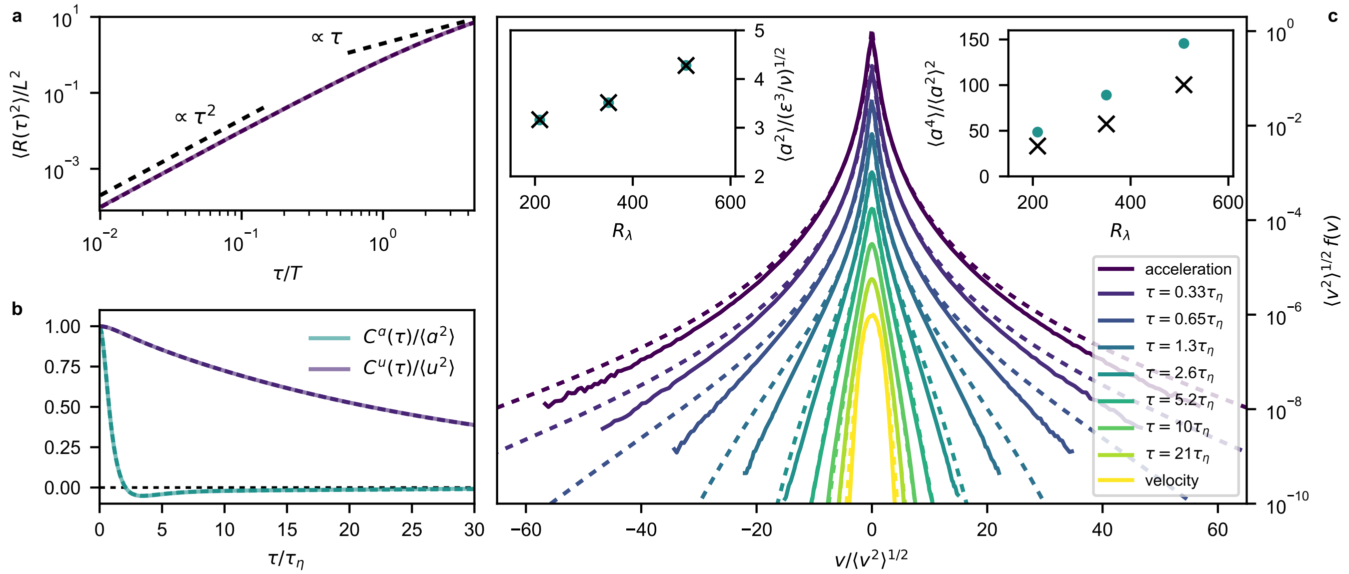

Supplementary Figure 2. **Predictions of acceleration, increment and velocity statistics** from our framework (dashed lines, dots) compared to DNS data (solid lines, crosses) for $\Theta = 0\tau_\eta$ at $R_\lambda \approx 350$. Second-order quantities like the mean squared displacement (**a**), the velocity and acceleration correlation functions (**b**) and the acceleration variance (**c**, left inset) are exactly captured by design of the model, and they do not depend on the choice of Θ. Acceleration and increment PDFs (**c**) agree significantly less well with the DNS data than for the case with coarse-graining, in particular for small time lags. The acceleration flatness (**c**, right inset) is systematically overestimated.

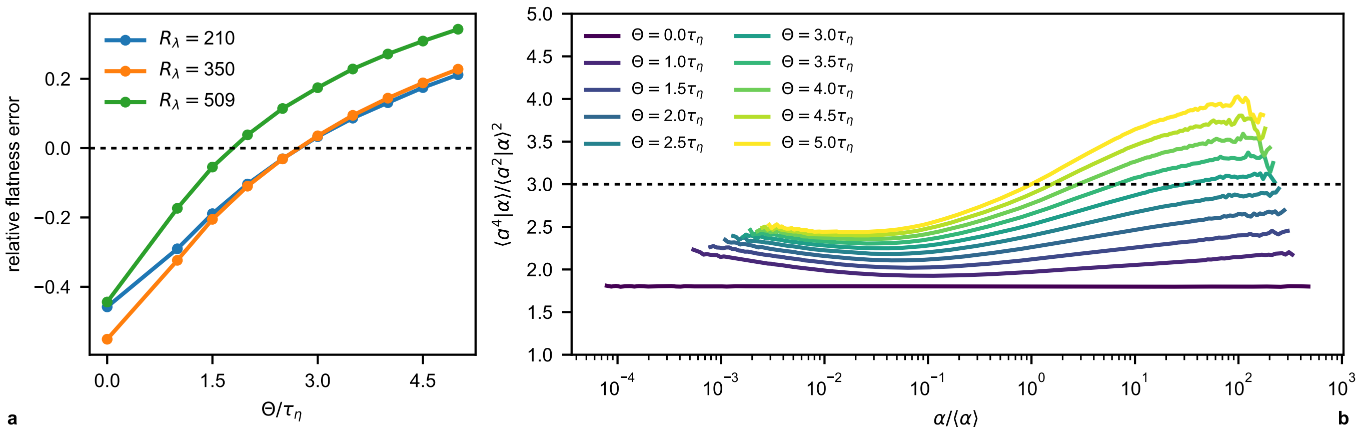

Supplementary Figure 3. **Acceleration flatness for different** Θ**.** **a** Relative prediction error of the acceleration flatness as a function of Θ, for three different simulations at varying Reynolds numbers. It is obtained by subtracting the predicted flatness of the framework from the flatness in the DNS and dividing by the DNS flatness. For small Θ, the flatness is overestimated. For large values, it is underestimated. **b** Conditional acceleration flatness on α for $R_\lambda \approx 350$, analogous to Fig. 3c in the main text. For $\Theta = 0$ a constant value of 9/5 is found, corresponding to uniform distributions. For larger Θ, the right tail of the curve changes most strongly. It reaches the Gaussian value three at Θ close to $3\tau_\eta$.

a convolution), the latter being very close to Gaussian in any case. We find that increment statistics are generally well modeled by our framework as long as the acceleration statistics are captured correctly. We therefore base our choice of Θ on the predicted acceleration flatness, a measure of the tails of the acceleration PDF. Supplementary Fig. 3a shows the relative error made by our framework in the prediction of the acceleration flatness compared to the DNS data as a function of Θ for the three different simulations at varying Reynolds numbers. For small Θ, the flatness is overestimated. For large Θ, it is underestimated. The optimal value of Θ, given by the zero-crossing, is close to $3\tau_\eta$ for our main data set at $R_\lambda \approx 350$, which is the value we use throughout this work unless noted otherwise. This choice of Θ can be further justified by looking at conditional statistics. Supplementary Fig. 3b shows the conditional acceleration flatness as a function of α and for various Θ for our main data set. The curve varies with Θ mainly in the

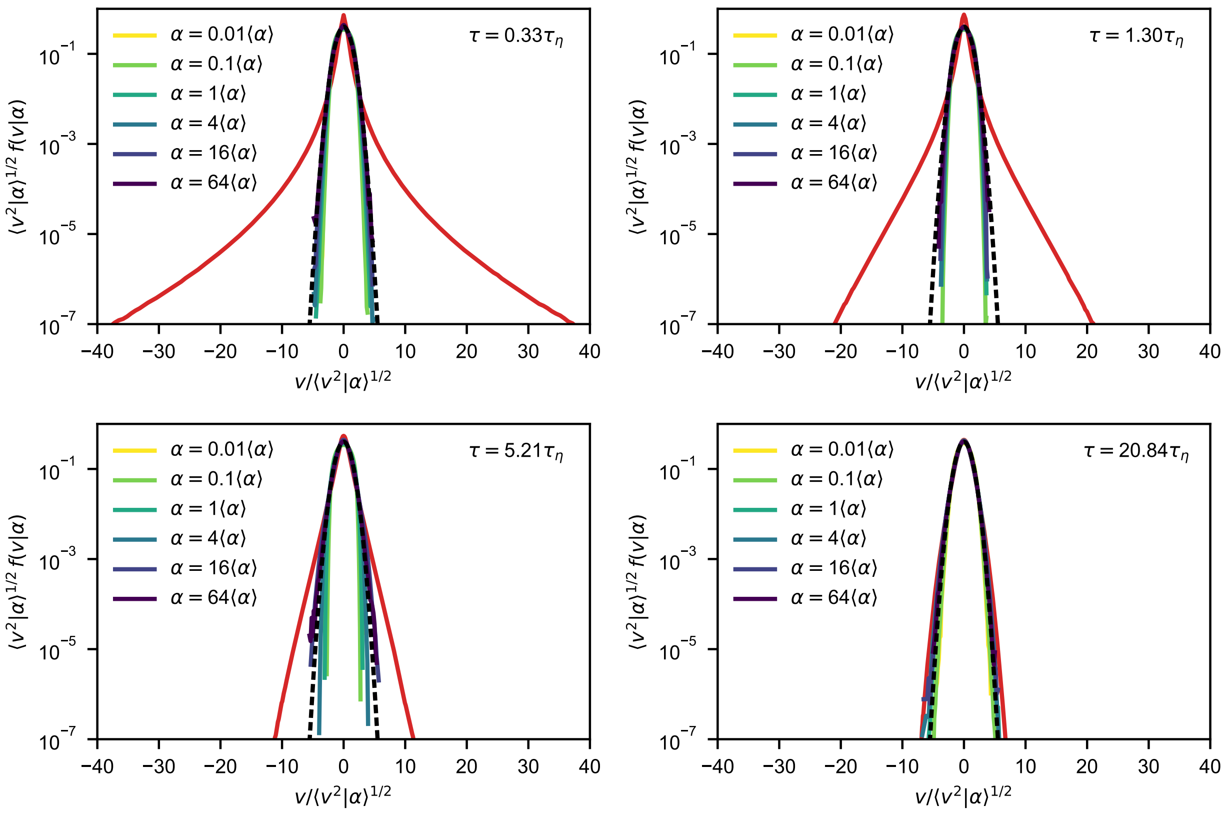

Supplementary Figure 4. **Conditional velocity increment PDFs** for $\Theta = 3\tau_\eta$ at $R_\lambda \approx 350$ and four different values of the time lag τ. While the unconditional PDF (red line) transitions from a heavy-tailed distribution for small time lags to an almost Gaussian distribution for large time lags, the conditional PDFs (colored lines) are close to Gaussian on all scales. A Gaussian distribution (black, dashed line) is plotted for comparison.

high-α regime, for which turbulence is intense and the flow changes quickly, so that a change of the coarse-graining time scale makes a significant difference. This right tail of the curve approaches the Gaussian value three most closely for our optimal $\Theta = 3\tau_\eta$. Another choice to be made regarding the coarse-graining is the filter shape. We have tested our results with respect to the filter shape and found that they are quite robust (not shown). Hence, we may simply choose a generic Gaussian filter.

SUPPLEMENTARY NOTE 2. CONDITIONAL VELOCITY INCREMENT STATISTICS

In this section, we provide additional results regarding the Gaussianity of conditional statistics on the coarse-grained squared acceleration α. In the main text, we restricted the discussion on the acceleration statistics, the most extreme example of non-Gaussian, heavy-tailed statistics in Lagrangian turbulence. However, non-Gaussianity can also be observed in the statistics of velocity increments, which transition continuously from heavy-tailed distributions for small time lags to almost Gaussian distributions for large time lags. Supplementary Fig. 4 shows the conditional velocity increment statistics on α for various time lags τ and various α along with their unconditional counterparts. On all scales, the conditional PDFs display a close-to-Gaussian form. Note that this is achieved using the single conditioning quantity α, where the coarse-graining time scale is fixed to $\Theta = 3\tau_\eta$. The coarse-grained squared acceleration α separates trajectories into sub-ensembles in which PDFs on all scales become almost Gaussian. This observation motivates our modeling of Lagrangian single-particle statistics as an ensemble of Gaussian time series. Furthermore, it explains why the model predictions of the increment PDFs, which are given by superpositions of Gaussian PDFs, agree closely with the DNS data (Fig. 5c).

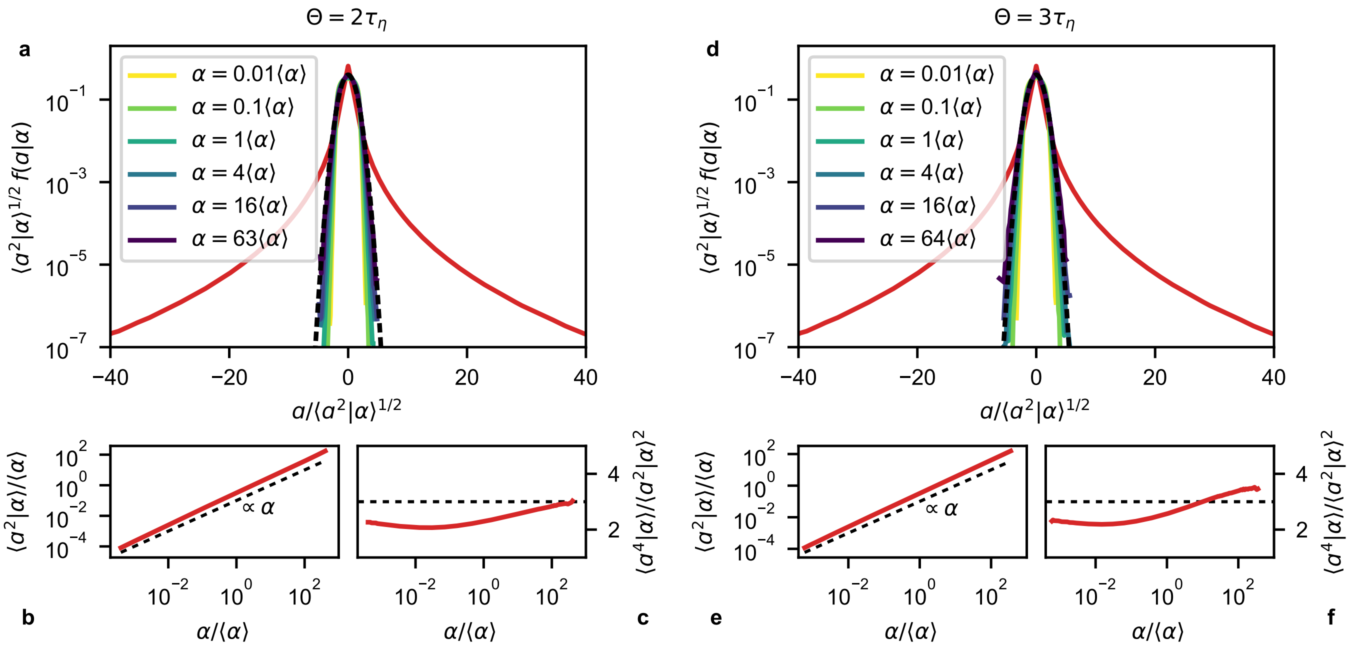

Supplementary Figure 5. **Conditional acceleration PDFs** at $R_\lambda \approx 509$ for $\Theta = 2\tau_\eta$ (left) and $\Theta = 3\tau_\eta$ (right). Colors correspond to the ones in Fig. 3. As in the main text, conditional PDFs are all close to Gaussian, for both values of Θ (**a**, **d**). The conditional second-order moment of the acceleration grows roughly like α (**b**, **e**). As in the main text, the flatness of the conditional acceleration is close to the Gaussian value three, for both values of Θ (**c**, **f**). However, the right tail of the curve approaches three more closely for the optimal choice $\Theta = 2\tau_\eta$.

SUPPLEMENTARY NOTE 3. RESULTS AT VARYING REYNOLDS NUMBER

In the main text, we focused on a well-resolved simulation at $R_\lambda \approx 350$. We have also confirmed that our framework generalizes well to other data sets at different Reynolds numbers. However, we find that the optimal choice of the coarse-graining time scale depends on the data set. As explained in the Supplementary Note 1, an optimal value of Θ can be determined by considering the error of the acceleration flatness prediction, shown in Supplementary Fig. 3a, and choosing Θ such that this error is minimized. Whereas the two simulations at $R_\lambda \approx 210$ and $R_\lambda \approx 350$ attain the correct flatness at Θ around $3\tau_\eta$, the figure suggests that for the simulation at $R_\lambda \approx 509$, Θ should be chosen around $2\tau_\eta$. Among the various data sets we analyzed (not all of them presented here), the optimal value of Θ varies, but we do not observe a clear trend (e.g. as a function of Reynolds number). We suspect that, besides the Reynolds number, other characteristics such as large-scale intermittency of the flow may play a role here, which is to be investigated in future work. However, we show in the following that our main results are robustly observed across various Reynolds numbers and choices of Θ, which is why the optimal choice of Θ is of overall minor importance. We here present results analogous to the Figs. 3 and 5c of the main text for the simulation at higher Reynolds number $R_\lambda \approx 509$. In order to illustrate the robustness with respect to the choice of the coarse-graining time scale, we compare results for $\Theta = 2\tau_\eta$ and $\Theta = 3\tau_\eta$. Supplementary Fig. 5 displays conditional acceleration statistics on α. For both values of Θ, they are indeed close to Gaussian. Supplementary Fig. 6 shows the predictions of acceleration, increment and velocity PDFs compared to the DNS data. Regarding the acceleration PDF, there is indeed an almost perfect collapse for the optimal $\Theta = 2\tau_\eta$. However, the overall agreement of the PDFs is comparable for $\Theta = 3\tau_\eta$. This illustrates that our results are both robust with respect to varying Reynolds number as well as different choices of the coarse-graining time scale Θ.

SUPPLEMENTARY NOTE 4. KINEMATIC VELOCITY INCREMENT PDF EQUATION

Here, we demonstrate that, by design, our framework is consistent with kinematic statistical evolution equations at the example of the PDF equation for Lagrangian velocity increments [1]

$$\partial_\tau f(v;\tau) = -\partial_v \left[\langle a|v;\tau\rangle f(v;\tau) \right] . \tag{1}$$

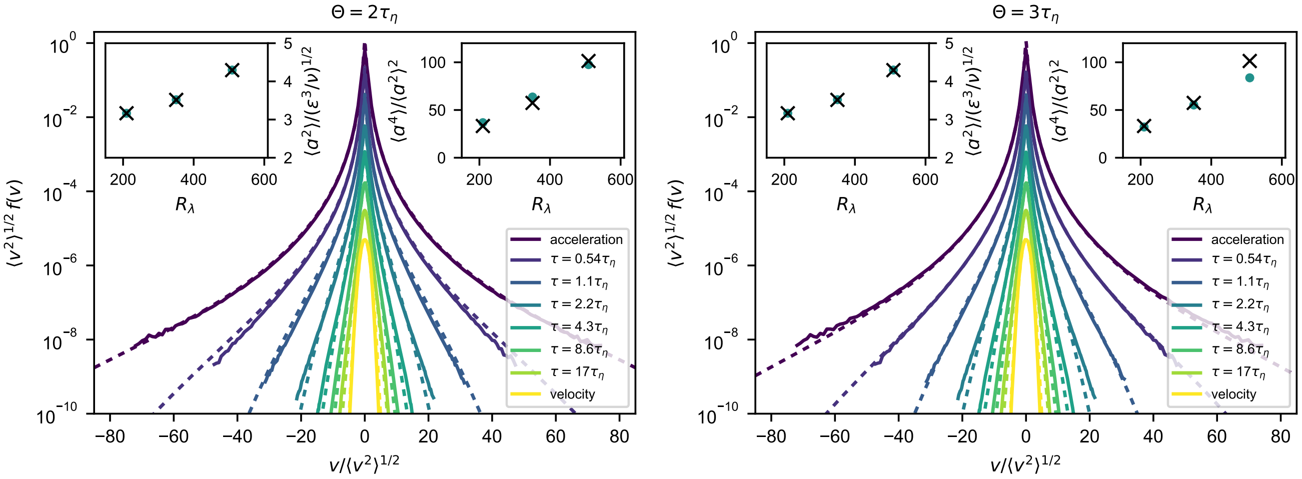

Supplementary Figure 6. **Predictions of acceleration, increment and velocity statistics** from our framework (dashed lines, dots) compared to DNS data (solid lines, crosses) at $R_\lambda \approx 509$ for $\Theta = 2\tau_\eta$ (left) and $\Theta = 3\tau_\eta$ (right). Both values of Θ provide reasonable predictions of the PDFs, illustrating the overall robustness with respect to the choice of Θ. The acceleration variance (left insets) agrees perfectly with the DNS value by design, independent of Θ. The acceleration flatness (right insets) is slightly underestimated for $\Theta = 3\tau_\eta$ at $R_\lambda \approx 509$. For $\Theta = 2\tau_\eta$ it is accurately predicted as ensured by our definition of the optimal Θ.

We use $v = u(t_0+\tau) - u(t_0)$ and evaluate the acceleration a at one of the endpoints. Both the increment PDF $f(v;\tau)$ and the mean acceleration conditioned on the increment $\langle a|v;\tau\rangle$ can be derived from the framework. For the latter, we need joint acceleration and increment statistics. The joint characteristic function,

$$\phi^{a,v}(\beta,\gamma;\tau) = \langle \exp(\mathrm{i}\beta a)\exp(\mathrm{i}\gamma v)\rangle, \tag{2}$$

can be calculated inserting $\vartheta(t) = -\beta \frac{\mathrm{d}}{\mathrm{d}t}\delta(t-t_0) + \gamma(\delta(t-t_0-\tau) - \delta(t-t_0))$ into the model given by Eq. (3) in the main text. All delta functions are then evaluated using integration by parts, yielding

$$\phi^{a,v}(\beta,\gamma;\tau) = \phi\left[\vartheta = -\beta \frac{\mathrm{d}}{\mathrm{d}t}\delta(t-t_0) + \gamma(\delta(t-t_0-\tau) - \delta(t-t_0))\right] \tag{3}$$

$$= \int_0^\infty \mathrm{d}\alpha\; f(\alpha)\exp\left(-\frac{1}{2}\left(-\beta^2 C''_\alpha(0) + \gamma^2 S_{2,\alpha}(\tau) - 2\beta\gamma C'_\alpha(\tau)\right)\right). \tag{4}$$

Recall that $S_{2,\alpha}(\tau) = 2C_\alpha(0) - 2C_\alpha(\tau)$ is the sub-ensemble second-order structure function. From this expression, we are able to calculate the conditional mean acceleration, which can be written as

$$\langle a|v;\tau\rangle = \frac{\int_{-\infty}^{\infty}\mathrm{d}a\; a\, f(a,v;\tau)}{f(v;\tau)}. \tag{5}$$

Here $f(a,v;\tau)$ denotes the joint PDF of acceleration and increments. The numerator of Supplementary Eq. (5) is obtained from the characteristic function in Supplementary Eq. (2) by deriving with respect to β:

$$\left[\partial_\beta \phi^{a,v}(\beta,\gamma;\tau)\right]_{\beta=0} = \left[\partial_\beta \langle \exp(\mathrm{i}\beta a)\exp(\mathrm{i}\gamma v)\rangle\right]_{\beta=0} \tag{6}$$

$$= \mathrm{i}\int_{-\infty}^{\infty}\mathrm{d}v\;\exp(\mathrm{i}\gamma v)\int_{-\infty}^{\infty}\mathrm{d}a\; a\, f(a,v;\tau). \tag{7}$$

This is an inverse Fourier transform, which may be canceled by a forward Fourier transform:

$$\langle a|v;\tau\rangle f(v;\tau) = \int_{-\infty}^{\infty}\mathrm{d}a\; a\, f(a,v;\tau) \tag{8}$$

$$= \frac{-\mathrm{i}}{2\pi}\int_{-\infty}^{\infty}\mathrm{d}\gamma\;\exp(-\mathrm{i}\gamma v)\left[\partial_\beta\phi^{a,v}(\beta,\gamma;\tau)\right]_{\beta=0}. \tag{9}$$

Inserting the explicit form of the characteristic function from Supplementary Eq. (4), we get:

$$\langle a|v;\tau\rangle f(v;\tau) = \int_0^\infty \mathrm{d}\alpha\; f(\alpha)C'_\alpha(\tau)\frac{-v}{S_{2,\alpha}(\tau)}\frac{1}{\sqrt{2\pi S_{2,\alpha}(\tau)}}\exp\left(-\frac{1}{2}\frac{v^2}{S_{2,\alpha}(\tau)}\right). \tag{10}$$

The increment PDF has been derived in the Methods section. By combining the increment PDF from Eq. (24) in the main text and the conditional mean acceleration from Supplementary Eq. (10), we can verify that the PDF equation is satisfied:

$$\partial_\tau f(v;\tau) \stackrel{(24)}{=} \int_0^\infty \mathrm{d}\alpha\; f(\alpha) C'_\alpha(\tau) \frac{S_{2,\alpha}(\tau) - v^2}{S_{2,\alpha}(\tau)^2} \frac{1}{\sqrt{2\pi S_{2,\alpha}(\tau)}} \exp\left(-\frac{1}{2}\frac{v^2}{S_{2,\alpha}(\tau)}\right) \tag{11}$$

$$\stackrel{(10)}{=} -\partial_v \left[\langle a|v;\tau\rangle f(v;\tau)\right] . \tag{12}$$

[1] Wilczek, M., Xu, H., Ouellette, N. T., Friedrich, R. & Bodenschatz, E. Generation of Lagrangian intermittency in turbulence by a self-similar mechanism. *New J. Phys.* **15**, 055015 (2013).
